# α-Cyclodextrin/Moringin Impacts Actin Cytoskeleton Dynamics with Potential Implications for Synaptic Organization: A Preliminary Transcriptomic Study in NSC-34 Motor Neurons

**DOI:** 10.3390/ijms26178220

**Published:** 2025-08-24

**Authors:** Agnese Gugliandolo, Luigi Chiricosta, Gabriella Calì, Patrick Rollin, Daniele Perenzoni, Renato Iori, Emanuela Mazzon, Simone D’Angiolini

**Affiliations:** 1 IRCCS Centro Neurolesi “Bonino-Pulejo”, Via Provinciale Palermo, Contrada Casazza, 98124 Messina, Italy; 2 Institute of Organic and Analytical Chemistry (ICOA), Université d’Orléans, UMR 7311, BP 6759, F-45067 Orléans, France; patrick.rollin@univ-orleans.fr; 3 Department of Food Quality and Nutrition, Research and Innovation Centre, Fondazione Edmund Mach (FEM), Via E. Mach 1, 38098 San Michele all’Adige, Italy; 4 Department of Innovative Technologies in Medicine & Dentistry, University “G. D’Annunzio” Chieti-Pescara, Via dei Vestini, 31, 66100 Chieti, Italy

**Keywords:** α-Cyclodextrin/Moringin, neuronal plasticity, cytoskeleton regulation, actin dynamics, transcriptomic analysis

## Abstract

α-Cyclodextrin/Moringin (α-CD/MOR) is an isothiocyanate showing neuroprotective and antioxidant properties. In this work, we studied in differentiated NSC-34 motor neurons cell line the molecular pathways activated following a treatment of 96 h with α-CD/MOR at different doses, namely 0.5, 5 and 10 μM. Taking advantage of comparative transcriptomic analysis, we retrieved the differentially expressed genes (DEGs) and we mapped DEGs to synaptic genes using the SynGO database. Then, we focused on the biological pathways in which they are involved. We observed that the prolonged treatment with α-CD/MOR significantly modulated biological processes and cellular components associated with synaptic organization. Interestingly, the KEGG pathway “Regulation of actin cytoskeleton” was overrepresented, alongside pathways related to synapses and axon guidance. Specifically, SPIA analysis indicated that the “Regulation of actin cytoskeleton” pathway was found to be activated with the highest dose of α-CD/MOR. Moreover, α-CD/MOR also modulated transcription factors involved in synaptic plasticity, such as *Creb1*. These results could indicate that α-CD/MOR can influence synaptic functions and organization, being involved in synaptic plasticity through the modulation of actin dynamics.

## 1. Introduction

Isothiocyanates (ITCs) are among the most recently studied phytocompounds. ITCs are a class of organic compounds formed as a result of the glucosinolates (GLs) hydrolysis through the enzyme myrosinase. GLs are present in different plants of the Cruciferae family (Brassicaceae), such as broccoli, mustard, cabbage, and cauliflower, and in the Moringaceae family. GLs are stable molecules in plant cells. The conversion from GLs to ITCs can occur as a defense mechanism after an injury to plant tissues (for instance, after the attack of predators or pathogens) [1]. In humans, after ingestion, GLs may be hydrolyzed by plant-derived myrosinase, and the resulting products are absorbed, while unmetabolized GL reach the colon, where they are processed by the gut microbiota [2].

Depending on the type of GLs, different ITCs are formed: glucoraphanin (GRA) and glucomoringin (GMG) produce sulforaphane (SFN) and moringin (MOR), respectively. In addition to the neuroprotective effects, ITCs can also exert anti-inflammatory, antioxidant, anticancer, and cardioprotective actions [3,4].

MOR is an ITC found in the plant *Moringa oleifera* [4]. *M. oleifera* is one of 12 species of the Moringaceae family. It is a tropical plant commonly called the “miraculous tree”. *M. oleifera* is widely used as a dietary supplement because of its rich nutritional composition, which includes vitamins, essential amino acids, minerals, and oleic acids [5]. It also contains bioactive substances that could support its therapeutic properties and offer positive effects on human health. One of the most studied bioactive substances is MOR. Indeed, several studies, both in vitro and in vivo, showed its antimicrobial [6], antioxidant [7,8], anti-inflammatory [9], antitumor [10], and neuroprotective [11,12] effects. In particular, MOR exerts protective effects in different models of neurodegenerative disorders [12,13,14].

Different phytochemicals were shown to promote neurite extension, synaptic transmission and formation, and the development of cytoskeletal structures, resulting in improved synaptic functionality [15]. The cytoskeleton is a highly dynamic structure that is composed of microtubules, actin filaments, and neurofilaments, and its rearrangement plays a key role in the regulation of synaptic plasticity. Specifically, actin dynamics is important in synaptic plasticity, modulating synapse organization and functions [16]. Synaptic stimulation rapidly changes actin dynamics, and many actin regulators play roles in neuronal functions. On the contrary, an abnormal synapse stabilization or pruning can lead to neurological complications. Accordingly, defects in the regulation of the actin cytoskeleton in neurons have been implicated in neurological disorders [17]. Cytoskeletal remodeling associated with synaptic functions is also relevant in neurodegenerative diseases and in aging-related processes [18]. Alterations of actin-binding proteins, which participate in actin cytoskeleton dynamics, could therefore represent a common feature of different synaptopathies, such as amyotrophic lateral sclerosis (ALS), frontotemporal dementia, and Alzheimer’s disease (AD) [19,20,21]. As an example, it is common to observe a compromised neuronal plasticity after traumatic events such as traumatic brain injury in which the cytoskeleton proteins undergo irreversible reactions that alter their functionality [22]. Synaptic plasticity also becomes impaired during aging, with changes occurring in the shape and density of dendritic spines [23]. Studies on AD post-mortem tissues and AD experimental models suggested that AD pathology negatively impacts actin cytoskeleton pathways. Indeed, actin polymerization influences clathrin-mediated endocytosis, which in turn controls Aβ production through the colocalization of the amyloid cascade proteins. So, the actin cytoskeleton represents a crossroad of pathways contributing to AD pathogenesis [19]. Motor neurons show long axonal projections, that require the integrity of neuronal cytoskeleton to maintain axonal stability, anterograde and retrograde transport, and signaling between neurons. For these reasons, protein aggregates containing cytoskeletal proteins, which represent a pathological feature of ALS, may alter the functions of neurons [20].

In this study, we investigated the effect of the prolonged treatment with the conjugated form α-cyclodextrin (CD)/MOR on the expression of genes involved in molecular pathways associated with synapses. Specifically, we differentiated NSC-34 cells, and treated them with the 0.5, 5 and 10 μM doses of α-CD/MOR for 96 h. The conjugated form α-CD/MOR showed an increased stability and solubility compared to MOR, which shows a low solubility in water [24]. We used transcriptomic analysis to investigate in more detail how this treatment can modulate gene ontology and pathways related to synaptic genes.

## 2. Results

### 2.1. Comparative Analysis of α-CD/MOR Against Control Group

**Figure 1:**
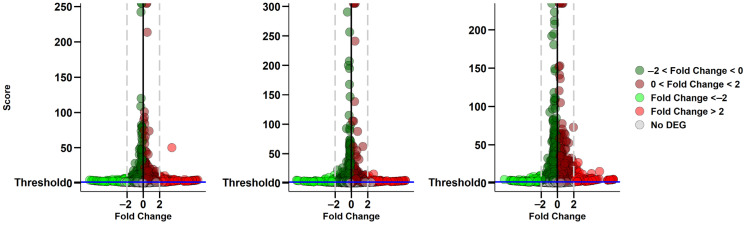
From left to right, the Volcano plots are related to the comparisons between CTRL vs. α-CD/MOR_0.5-96_, CTRL vs. α-CD/MOR_5-96,_ and CTRL vs. α-CD/MOR_10-96_. The overrepresented DEGs (more expressed in α-CD/MOR) are highlighted in red if they have a fold change higher than 2 and in dark red otherwise. On the other hand, the downregulated DEGs (more expressed in CTRL), are depicted in green when the fold change is lower than −2, and in dark green if it is not. Non-statistically significant genes are represented in gray. The fold change is computed as the log_2_(α-CD/MOR/CTRL). The score is computed as the log_10_(*p*-adjusted). The threshold is set to the *p*-adjusted 0.05.

The comparative analysis of the control (CTRL) group versus α-CD/MOR-treated samples for 96 h with 0.5 μM (CTRL vs. α-CD/MOR_0.5-96_), 5 μM (CTRL vs. α-CD/MOR_5-96_), or 10 μM (CTRL vs. α-CD/MOR_10-96_) is presented item by item in Figure 1 using Volcano plots.

The amount of differentially expressed genes (DEGs) in the three groups and the number of up- or downregulated genes were of the same order of magnitude and increased proportionally to the used dose of α-CD/MOR. In detail, 5005, 5320, and 5964 DEGs were found in CTRL vs. α-CD/MOR_0.5-96_, CTRL vs. α-CD/MOR_5-96,_ and CTRL vs. α-CD/MOR_10-96,_ respectively (Tables S1–S3). Also, the amount of upregulated and downregulated DEGs was 2524 and 2481 for CTRL vs. α-CD/MOR_0.5-96_, 2611 and 2709 for CTRL vs. α-CD/MOR_5-96,_ and 2957 and 3007 for CTRL vs. α-CD/MOR_10-96_, respectively.

### 2.2. α-CD/MOR Modulated Synaptic Ontologies

The focus of our study was to observe in differentiated motor neuronal NSC-34 cells the effects of the prolonged exposure to α-CD/MOR on synaptic genes. In this sense, we took advantage of the curated SynGO database [22] accessible at https://syngoportal.org (30 June 2025), to associate our DEGs to a specific neurological context. Since SynGO is curated on human genes, it provides the mapping tool for alias conversion from other species. After mapping, SynGO was able to provide an association for 4872 DEGs in CTRL vs. α-CD/MOR_0.5-96_, 5196 DEGs in CTRL vs. α-CD/MOR_5-96_, and 5815 DEGs in CTRL vs. α-CD/MOR_10-96_. In particular, the DEGs mapped on 302 terms (Tables S4–S6). For each term, the q-value was obtained using false discovery rate as post hoc correction. We set the threshold of 0.05 for the q-value to discriminate statistically significant terms. As shown in Table 1, eight terms were under the threshold, and they were three biological process (BP) and five cellular component (CC) terms.

**Figure 2:**
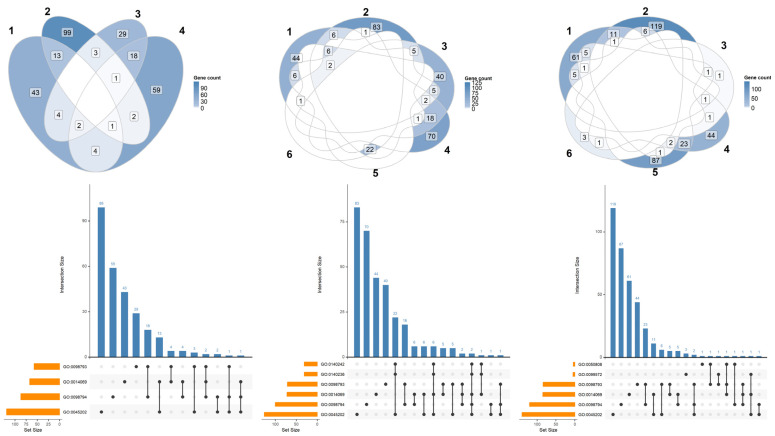
The comparisons CTRL vs. α-CD/MOR_0.5-96_, CTRL vs. α-CD/MOR_5-96_, and CTRL vs. α-CD/MOR_10-96,_ from left to right, are depicted using Venn diagram in the upper panels and the relative upset distributions in the bottom panels. The scale color is related to the number of genes that are included in each term. Regarding the Venn plots, the terms referred for CTRL vs. α-CD/MOR_0.5-96_ to 1-GO:0045202, 2-GO:0098793, 3-GO:0098794, 4-GO:0014069; for CTRL vs. α-CD/MOR_5-96_ to 1-GO:0045202, 2-GO:0098793, 3-GO:0098794, 4-GO:0014069, 5-GO:0140236, 6-GO:0140242; and for CTRL vs. α-CD/MOR_10-96_ to 1-GO:0045202, 2-GO:0098793, 3-GO:0098794, 4-GO:0099572, 5-GO:0014069, 6-GO:0050808. In the bottom panel, the barplots shows the number of genes in each combination of terms while the black dot plot highlights the combination of term for each bar.

To inspect how the DEGs are distributed among the overrepresented terms, we computed the Venn plots in Figure 2 along with upset distribution for each comparison. No comparison showed any DEG taking place in all the terms. Most DEGs were specific to the particular term, and in some cases, they are shared among the terms. In this sense, the terms were quite specific and independent from each other for each comparison. In detail, among the significant terms of the Gene Ontology dictionary, CTRL vs. α-CD/MOR_0.5-96_ included four terms: synapse (GO:0045202), presynapse (GO:0098793), postsynapse (GO:0098794), and postsynaptic density (GO:0014069). CTRL vs. α-CD/MOR_5-96_ included six terms: synapse (GO:0045202), presynapse (GO:0098793), postsynapse (GO:0098794), postsynaptic density (GO:0014069), translation at presynapse (GO:0140236), and translation at postysnapse (GO:0140242). CTRL vs. α-CD/MOR_10-96_ included six terms: synapse (GO:0045202), presynapse (GO:0098793), postsynapse (GO:0098794), postsynaptic specialization (GO:0099572), postsynaptic density (GO:0014069), and synapse organization (GO:0050808). Thus, the general terms GO:0045202, GO:0098793, GO:0098794, and GO:0014069 were shared among all the comparisons.

**Figure 3:**
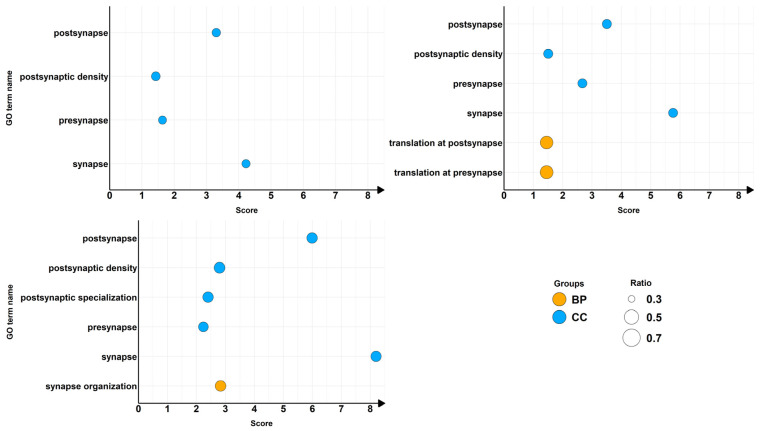
Overrepresented terms of DEGs mapped on SynGO related to CTRL vs. α-CD/MOR_0.5-96_ (**upper left** panel), CTRL vs. α-CD/MOR_5-96_ (**upper right panel**) and CTRL vs. α-CD/MOR_10-96_ (**bottom panel**). The terms highlighted in the plot have a q-value lower than 0.05. The score is obtained as −log_10_ q-value. The orange bubbles represent biological process terms, while the blue bubbles represent cellular component terms. The size of the bubbles is obtained as the ratio of the DEGs mapped in the terms over the number of genes included in the term.

We then inspected, as shown in Figure 3, the different categories to which each overrepresented term belongs and the relative amount represented as the ratio of the DEGs mapped in the terms over the number of genes included in the term.

### 2.3. α-CD/MOR Modulated Synaptic Pathways

**Figure 4:**
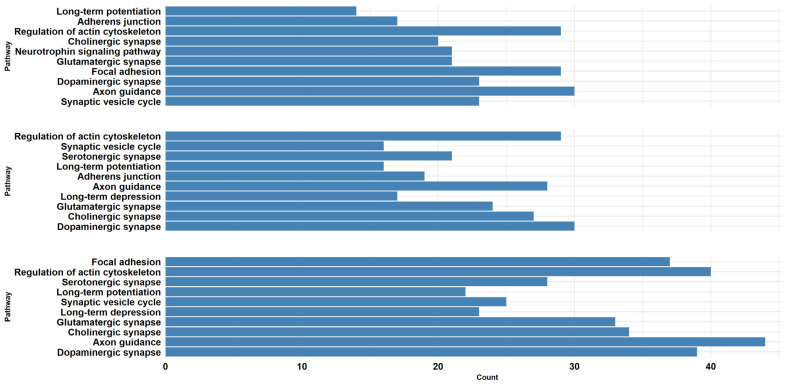
From top to bottom, the comparisons CTRL vs. α-CD/MOR_0.5-96_, CTRL vs. α-CD/MOR_5-96_ and CTRL vs. α-CD/MOR_10-96_ are depicted using barplots. For each comparison, we represented the 10 pathways, sorted by lowest q-value, obtained with KEGG overrepresentation of DEGs mapped on SynGO. The count refers to the number of DEGs observed in the pathway.

Then, for each comparison, we performed the pathway overrepresentation analysis of the DEGs mapped on SynGO using the KEGG database [25], considering the same threshold of q-value (0.05) (Tables S7–S9). Among all the pathways represented in KEGG, we kept all the pathways related to our experiment that are included under the categories “Cell growth and death”, “Cell motility”, “Cellular community—eukaryotes”, “Development and regeneration”, “Nervous system”, and “Neurodegenerative disease”. Finally, CTRL vs. α-CD/MOR_0.5-96_ and CTRL vs. α-CD/MOR_5-96_ counted 21 pathways, while CTRL vs. α-CD/MOR_10-96_ counted 26 pathways. In order to obtain a better representation, we selected the 10 pathways with the lowest q-values from each comparison group and represented them in Figure 4. The majority of the pathways were common between the comparisons. Notably, the “Axon guidance” and “Regulation of actin cytoskeleton” pathways were among those with more genes in all the comparisons.

Given the importance of the pathway “Regulation of actin cytoskeleton” in synapse function and organization, we evaluated the activation state of this pathway in the different comparisons using SPIA. SPIA analysis revealed that this pathway was activated only in the comparison CTRL vs. α-CD/MOR_10-96_, while in the others, it was inhibited.

### 2.4. α-CD/MOR-Treated Cells Expressed Neuronal Markers

**Figure 5:**
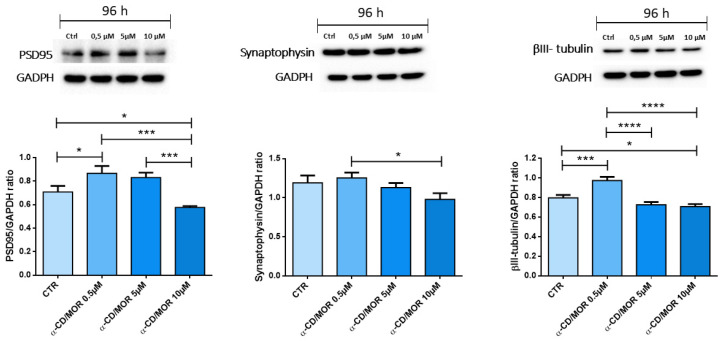
Western blot analysis of the neuronal markers PSD95, synaptophysin, and βIII-tubulin. Treatment with α-CD/MOR_0.5-96_ significantly increased PSD95 and βIII-tubulin, while α-CD/MOR_10-96_ decreased their levels. α-CD/MOR_10-96_ did not significantly change synaptophysin protein levels compared to control. * *p* < 0.05; *** *p* < 0.001; **** *p* < 0.0001.

We performed the Western blot analysis of neuronal markers PSD95, synaptophysin, and βIII-tubulin (Figure 5).

PSD95 and βIII-tubulin significantly increased in α-CD/MOR_0.5-96_, while their protein levels decreased in the α-CD/MOR_10-96_ group compared to the control. Synaptophysin was expressed in all groups and showed no significant differences compared to the control.

### 2.5. Integration of Synaptic Genes into Cytoskeleton Regulation

Interaction networks were generated for each comparison using the STRING database and analyzed in Cytoscape.

The initial network consisted of 574 nodes for the CTRL vs. α-CD/MOR_0.5-96_ comparison, 644 nodes for the CTRL vs. α-CD/MOR_5-96_ comparison, and 726 nodes for the CTRL vs. α-CD/MOR_10-96_ comparison. After removing proteins not connected to the main network, the nodes were reduced to 372 for the CTRL vs. α-CD/MOR_0.5-96_ comparison, 411 for the CTRL vs. α-CD/MOR_5-96_ comparison, and 494 for the CTRL vs. α-CD/MOR_10-96_ comparison.

**Figure 6:**
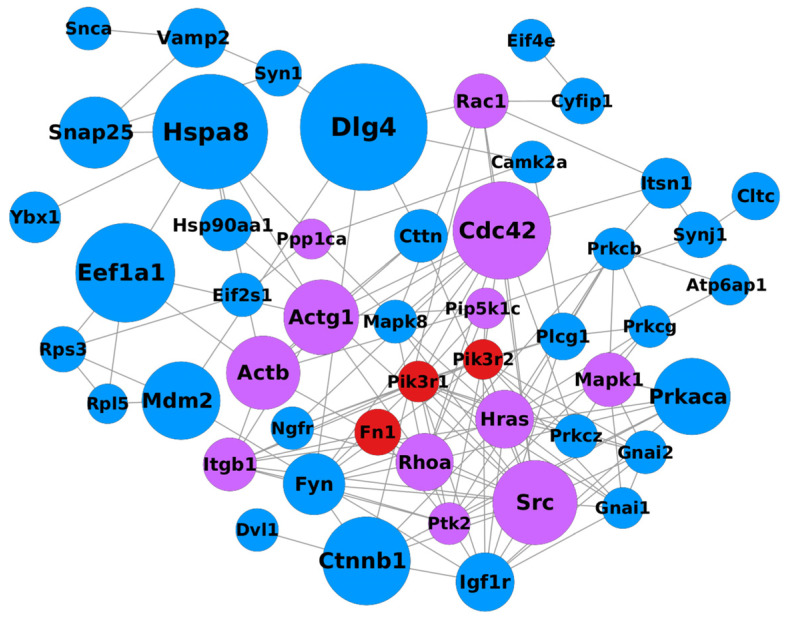
The network highlights nodes with betweenness centrality above the 90th percentile in the CTRL vs. α-CD/MOR_10-96_ comparison. Inside each node is reported the name of the related protein. The size of each node is proportional to its betweenness centrality. Blue nodes are related to proteins mapped in SynGO, red nodes are related to proteins involved in the “Regulation of actin cytoskeleton” KEGG pathways, while purple nodes are related to proteins mapped in SynGO that also take part in the “Regulation of actin cytoskeleton” pathway.

Subsequently, for each network, proteins with a betweenness centrality above the 90th percentile were plotted. The final filtered network for the CTRL vs. α-CD/MOR_10-96_ comparison is reported in Figure 6, while the networks for the comparisons CTRL vs. α-CD/MOR_0.5-96_ and CTRL vs. α-CD/MOR_5-96_ were reported in Figures S1 and S2, respectively. All the tables with the information related to all the nodes of the different networks are provided in Supplementary Table S10.

### 2.6. Transcription Factors Involved in α-CD/MOR Effects and Analysis of DEGs Associated with Neurodegenerative, Neurological, and Neuropsychiatric Diseases

In order to identify the transcription factors that could be involved in the modulation of the transcriptomic profile of the inspected groups, we used the web tool Enrichr (https://maayanlab.cloud/Enrichr/, accessed on 5 August 2025). Specifically, we took advantage of the ChEA database with default parameters to highlight the overrepresented transcription factors. The chosen threshold of 0.05 for *p*-value, adjusted by post hoc correction, showed 588 transcription factors for α-CD/MOR_0.5-96_, 624 transcription factors for α-CD/MOR_5-96_, and 656 transcription factors for α-CD/MOR_10-96_ (Supplementary Table S11).

Additionally, the same tool was used to identify overrepresented disorders in which our identified DEGs were involved for each comparison with the same statistical significance. In particular, the database DisGeNET showed 150 diseases for α-CD/MOR_0.5-96_, 178 diseases for α-CD/MOR_5-96_, and 350 diseases for α-CD/MOR_10-96_ (Supplementary Table S12). Among them, 14 diseases for α-CD/MOR_0.5-96_, 18 diseases for α-CD/MOR_5-96_, and 35 diseases for α-CD/MOR_10-96_ were related to neurodegenerative, neurological, and neuropsychiatric diseases.

**Figure 7:**
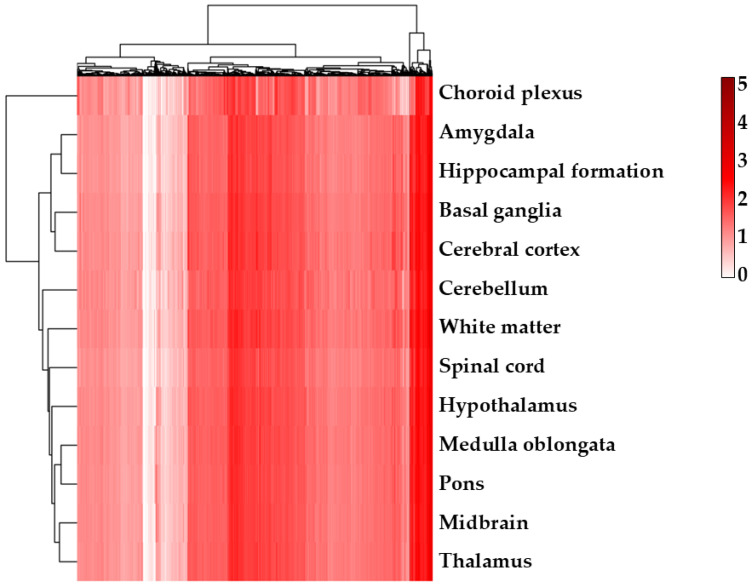
Heatmap with expression level of transcripts identified as DEGs in CTRL vs. α-CD/MOR_10-96_ comparison in the different brain regions of the Human Protein Atlas. Each line of the dendogram on *x*-axis represent a DEG. Conversely, each line on *y*-axis stands for brain region.

Given the amount of neurological disorders found as overrepresented, we observed for each comparison how the expression level of transcripts identified as DEGs in our experiments were distributed among the different brain regions in the Human Protein Atlas to identify possible cross-interactions (Figure 7; Supplementary Figures S3 and S4 for CTRL vs. α-CD/MOR_0.5-96_ and CTRL vs. α-CD/MOR_5-96_ comparisons, respectively).

## 3. Discussion

α-CD/MOR demonstrated several protective effects and neuroprotective properties. In a previous study, we showed that α-CD/MOR at 10 μM for 96 h exhibited antioxidant and neuroprotective effects, modulating the expression of NRF2 and its interactors in differentiated motor neuron NSC-34 cells [26]. Here, we evaluated the effects of α-CD/MOR on synaptic genes in differentiated NSC-34 cells after a prolonged exposure. For this reason, we treated differentiated NSC-34 cells for 96 h with different α-CD/MOR concentrations, namely 0.5, 5 and 10 μM. SynGO database shows a significant overrepresentation of both biological process (BP) and cellular component (CC) terms in our comparisons, except for the CTRL vs. 0.5 μM α-CD/MOR. Indeed, in comparison CTRL vs. α-CD/MOR_0.5-96_, only CC terms were overrepresented. The different terms shared few genes, while the majority were unique for each term. In particular, the CC “synapse” was significantly overrepresented in all the groups, highlighting that α-CD/MOR was able to modulate synaptic genes at all the concentrations. Interestingly, even if “presynapse” was overrepresented in all the comparisons, the postsynaptic compartment was more interested by α-CD/MOR effects. Indeed, the “postsynapse” term had a higher number of DEGs. Moreover, the terms “postsynapse” and “postsynaptic density” were overrepresented in all comparisons, while “postsynaptic specialization” was overrepresented in comparison CTRL vs. 10 μM α-CD/MOR. These results indicated that α-CD/MOR may influence synapse organization, and especially postsynaptic region, influencing the spatial and functional organization of postsynaptic proteins, such as anchoring and scaffolding molecules, neurotransmitter receptors, enzymes, and cytoskeletal components. In accordance with that, previous reports indicated that *M. oleifera* extracts promoted synaptic plasticity [27,28].

Interestingly, KEGG analysis also revealed that some pathways were significantly overrepresented in all the comparisons, such as synaptic pathways, “long-term potentiation”, “Axon guidance”, and “Regulation of actin cytoskeleton”. Interestingly, “Regulation of actin cytoskeleton” pathway was one of the most represented ones in all comparisons in terms of the number of included genes. This result is in accordance with GO results which indicated that α-CD/MOR modulated genes involved in synapse modulation and organization. Cytoskeleton plays a crucial role in cellular physiology, especially in processes such as cell motility, cell shape, and intercellular communication. In the context of motor neurons, the regulation of the cytoskeleton becomes even more critical, as it is essential for the growth, extension, and branching of neurites. These processes are fundamental for the formation of neuronal connections and the promotion of synaptic plasticity. Specifically, the synaptic actin cytoskeleton is a key element in synapse stability. Actin has a main role in regulating synapse structure and functions, such as spine morphology, post-synaptic density organization, long-term potentiation, anchoring, and trafficking of post-synaptic receptors. Then, synaptic plasticity induces remodeling of the actin cytoskeleton at the synapse, including pre- and post-synaptic compartments [16].

Given that “Regulation of actin cytoskeleton” emerged from the KEGG analysis as one of the pathways with the highest number of genes, these results indicate that the regulation of the actin cytoskeleton plays a central and fundamental role in the modulation of synaptic organization mediated by α-CD/MOR. Moreover, the pathway “Regulation of actin cytoskeleton” represents a base for all other pathways, given that actin cytoskeleton influences all others, including synapses, axon guidance, LTP, and junctions. Among these, the “Axon guidance” pathway was one of the bigger pathways overrepresented in all comparisons. Axon outgrowth is guided by growth cones, high motility structures enriched in filamentous actin at the axon distal tips. Growth cones can scan the environment using their F-actin protrusions in order to sense guidance cues and respond to them controlling actin dynamics. Actin dynamics in growth cones steers the axon towards attractants and away from repellents [29]. Among the main guidance cues that induce actin cytoskeletal modifications are slit and its Robo receptors, semaphorins and their plexin and neuropilin receptors, and ephrin and its Eph receptors. They initiate a cascade of events to modulate the growth cone membrane and the cytoskeleton to modulate axon growth and guidance [30].

**Figure 8:**
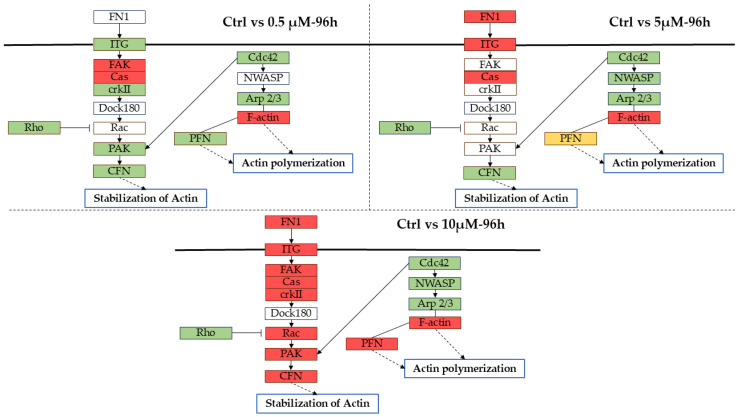
“Regulation of actin cytoskeleton” (mmu04810) KEGG pathway at different α-CD/MOR doses. A schematic representation of a comparison of the main DEGs involved in the pathway related to the regulation of actin cytoskeleton for CTRL vs. α-CD/MOR 0.5 μM-96 h, CTRL vs. α-CD/MOR 5 μM-96 h, CTRL vs. α-CD/MOR 10 μM-96 h. Red background is related to upregulated proteins while the green one is related to downregulated proteins. The yellow background is present to highlight a protein made by both upregulated and downregulated genes. Solid line represent direct connection between protein while dashed one highlight undirected activation of processes.

Interestingly, the pathway “Regulation of actin cytoskeleton” was overrepresented at all the tested concentrations. However, the SPIA analysis revealed that it was activated only with the highest α-CD/MOR concentration, suggesting that lower ones were not sufficient to exert this action. Indeed, some key genes involved in actin dynamics were DEGs or upregulated only at this concentration (Figure 8). For this reason, we focused on the concentration of 10 µM α-CD/MOR.

Integrin receptors make essential contributions to neurite outgrowth and axon elongation. Activated integrins engage extracellular matrix components allowing the growth cone to form contact points, which link the extracellular substrate to dynamic intracellular protein complexes [31]. In our study, we observed that the genes *Itga2*, *Itgb1*, and *Itgb3*, encoding the different integrin subunits, were upregulated, while *Itga5* was downregulated. Among them, *Itgb1,* and *Itgb3* are directly correlated to the nervous system. Specifically, Itgb1 plays an important role in the formation of intraneuronal connections, promoting neurite growth, axon guidance, and synapse formation and maturation [32]. Itgb3 is essential for a normal dendritic morphology in pyramidal neurons [33].

Integrin-mediated signaling recruits the complex Cas/CrkII/FAK. In our study, *Crk* and *Ptk2* were involved in its formation, and they were upregulated. The protein Cas forms a complex with non-receptor focal adhesion kinases (FAK) and SRC family kinases (SFKs), particularly the CrkII protein, encoded by *Crk*. In vitro studies demonstrated the important role of the Cas protein in mediating the integrin signal during neuronal development and the axon guidance activation [34]. The protein FAK, encoded by *Ptk2*, is broadly expressed in the mammalian brain and highly enriched in neuronal growth cones. In vitro and in vivo studies showed that FAK expression positively modulates neurite outgrowth and synaptic plasticity. It is also involved in the formation and preservation of long-term spatial memory [35].

Rho family GTPases are fundamental regulatory molecules, linking surface receptors with actin cytoskeleton organization. Specifically, Rho GTPases are involved in the regulation of neuronal morphology, including dendritic arborization, spine morphogenesis, growth cone development, and axon guidance [36]. RhoA, Rac, and Cdc42 were indicated as important regulators of axonal and dendrite morphogenesis. Rho proteins are known to inhibit neurite extension; in contrast, Cdc42 and Rac are positive regulators of neurite outgrowth and dendritic spine formation. In particular, they promote protrusions through actin filament assembly [37]. Studies confirmed an important role of Rac1 in neurite outgrowth and axonal pathfinding, as well as neuronal migration [36]. In our study, only *Rac1* was upregulated, while *Rhoa* and *Cdc42* were downregulated in CTRL vs. α-CD/MOR_10-96_. Interestingly, *Rac1* was upregulated only in CTRL vs. α-CD/MOR_10-96_, while it was not differentially expressed with lower α-CD/MOR concentrations. A study on motor neurons showed that reduced Rac activity is associated with increased death. In addition, it showed that Rac is downregulated in patients with sporadic ALS. On the other hand, RhoA expression may cause growth cone collapse by inhibiting neurite extension through stress fiber formation. Several studies suggested that an imbalance between the expression of RhoA and Rac could be one of the contributing factors to ALS [38]. Interestingly, Rho family GTPases play a role also in neuron death and survival. Specifically, Rho indirectly inactivates pro-survival proteins suppressing neuronal survival. In contrast, Rac enhances neuronal survival also via the activation of PAK [36].

An effector of *Rac1*, *Pak2* gene, encoding for Pak protein, was upregulated only in the comparison CTRL vs. α-CD/MOR_10-96_. Pak2 belongs to the group I Paks along with Pak1 and Pak3, and they are very similar in structure. Several studies showed that Paks, particularly PAK1, are involved in neuronal polarization, differentiation, and migration. A study in PAK1 knockout mice showed a reduction in the number of pyramidal neurons and impaired neuronal migration [39]. Very little is known about *Pak2*, but their similarities in sequence and structure suggest that both proteins perform similar functions in cytoskeleton regulation. Moreover, Pak2 haploinsufficiency caused a decrease in synapse densities, defective long-term potentiation, and autism-related behaviors in mice, but also alterations in actin polymerization [40].

The ability of cofilin to modulate actin cytoskeleton rearrangements confers an important role in neurite outgrowth and guidance. Indeed, growth cone motility is based on the dynamic assembly and disassembly of actin filaments [41]. The function of cofilin is spatially and temporally regulated within the growth cone. In fact, in the anterior part of the growth cone, actin polymerizes, enabling forward movement. In contrast, in the posterior part of the growth cone, actin filaments undergo depolymerization to ensure forward movement [42]. Cofilin is capable of regulating actin dynamics, and in this study, *Cfl1* gene was upregulated. At low cofilin/actin ratios, cofilin severs F-actin, increasing the ADP-actin monomer dissociation rate. At high cofilin/actin ratios, cofilin stabilizes F-actin or even the nucleation of new filaments. Inactive phosphorylated cofilin does not significantly bind to F-actin, and actin severing or depolymerization is low [43].

Profilin is a monomeric (G-)actin-binding protein needed in all non-muscle cells to maintain a G-actin pool necessary for the fast actin dynamics of these cells. More isoforms exist in mammals. Profilin 1 is ubiquitously expressed [44]. It binds G-actin in a 1:1 ratio, positively regulating actin polymerization. Profilin1, encoded by the *Pfn1* gene, was upregulated in our study. It is an important regulator of synaptic plasticity thanks to its contribution to actin dynamics and cytoskeletal integrity and is needed in synaptogenesis [45].

Arp2/3 complex genes *Actr2* and *Arpc2* were downregulated. A study demonstrated that inhibiting the Arp2/3 complex in neurons has no effect on the dynamics of actin in the growth cone and, as a result, has no negative impact on neurite formation [46]. The genes encoding for actin (*Actb* and *Actg1*) were upregulated in CTRL vs. α-CD/MOR_10-96_.

Interestingly, only at the concentration of 10 μM α-CD/MOR, after 96 h, key genes involved in actin dynamics, such as *Rac1*, *Cfl1*, and *Pak2*, were upregulated. Instead, at lower concentrations, they were downregulated or not differentially expressed.

We also constructed an interaction network among the DEGs related to actin remodeling to highlight how they integrate with synaptic components and identify central regulatory nodes. It is not surprising that synaptic genes that also take part in the pathway “Regulation of actin cytoskeleton” represented important nodes of the network, such as Cdc42, Actg1, and Actb.

We evaluated the expression of neuronal markers using Western blot. βIII-tubulin is one of the earliest markers of neuronal differentiation of both the central and peripheral nervous systems. *Tubb3* expression reaches a peak during axonal guidance and neuronal maturation, and its levels decreased in the central nervous system with maturity, while they were maintained high in the peripheral nervous system [47]. In our study, *Tubb3* expression was downregulated with the concentration 10 µM α-CD/MOR and Western blot analysis confirmed the reduced protein levels. A study showed that reduced *Tubb3* levels accelerated microtubule growth in axons and dendrites, and *Tubb3* knockdown induced a specific upregulation of *Tubb4* gene expression [48]. These data are in line with our transcriptomic results, in which *Tubb3* downregulation is associated with *Tubb4b* upregulation in cells treated with 10 µM α-CD/MOR.

PSD-95 is a major component of the post synaptic density, an electron-dense specialization of excitatory postsynaptic membranes containing high concentrations of glutamate receptors and associated signaling and cytoskeletal proteins. PSD95 interacts with postsynaptic glutamate receptors and is important for their synaptic signaling [49]. PSD-95 acts as a scaffolding protein during synaptogenesis, regulates synaptic maturation [50], and maintains excitatory synapse balance [51]. However, PSD95 also has a role in excitotoxicity triggered by NMDARs. Indeed, in cultured cortical neurons, the suppression of PSD95 expression attenuated excitotoxicity caused by NMDA receptors [52]. PSD95 gene silencing delayed cell death in postischemic rat hippocampus [53]. A study also showed that PSD95-gene-specific siRNAs may relieve neuropathic pain [54]. In this study, we found a decrease in PSD95 after a 96 h treatment with the concentration 10 µM α-CD/MOR, in accordance with the transcriptomic analysis that evidenced the downregulation of the gene *Dlg4* encoding for PSD95. Its decrease may indicate the maintenance of excitatory versus inhibitory balance and the absence of excitotoxicity, that is a hallmark of neurodegeneration. Then, α-CD/MOR may also be helpful in those conditions characterized by excitotoxcity.

Synaptophysin is a presynaptic protein and the second most abundant cargo on the synaptic vesicles. Synaptophysin is involved in the formation of synaptic vesicles and their exocytosis and drives the synapsis formation. Synaptophysin seems to coordinate the retrieval of synaptobrevin 2 during synaptic vesicle endocytosis [55]. In our study, synaptophysin is expressed in all groups; however, there were no significant differences compared to control, confirming transcriptomic analysis. This result is in line with GO results, which indicated a less prominent role of α-CD/MOR at the presynaptic level.

We also identified upstream transcription factors known to regulate gene expression of our DEGs to deeply evaluate the mechanism of action of α-CD/MOR. Among the most significant transcription factors, we found the members of the cAMP response element binding protein family, CREB and CREM [56]. CREB is a key component in diverse physiological processes, including nervous system development, neuronal survival, postnatal hippocampal neurogenesis, synaptic plasticity, and neurite outgrowth, and it has neuroprotective properties [57]. Also the transcription factor YY1 was identified, involved in proliferation, differentiation, and apoptosis; specifically it plays a role in neural development, neuronal function, and myelination [58]. A recent study evidenced that Yy1 controls murine cerebral cortex development in a stage-dependent manner. Specifically, it maintains the proliferation and survival of neural progenitor cells at early stages of brain development, while the dependence on Yy1 decreases during corticogenesis [59]. Also, Runx2 was among the significant transcription factors. A study indicated that Runx2 may be involved in neurite outgrowth, Schwann cell differentiation, and migration after sciatic nerve injury [60]. Interestingly, in our study, the transcription factors *Creb1* and *Runx2* were upregulated, suggesting that another mechanism that mediates α-CD/MOR effects on synaptic genes may be associated with the upregulation of these transcription factors.

Interestingly, DEGs modulated by α-CD/MOR were shown to be involved in different neurological, neurodegenerative, and neuropsychiatric conditions and to be expressed in different brain areas. This result may be of interest and support the use of α-CD/MOR as an integrative therapy in different synaptopathies associated with cytoskeletal alterations.

Our study is in accordance with previous studies which demonstrated ITCs effects on synaptic plasticity and cytoskeleton remodeling. SFN, through the modulation of Nrf2, enhanced white matter plasticity, improving the pyramidal tract plasticity and regeneration of oligodendrocytes after ischemic stroke [61]. SFN administration during postnatal brain development in mice enhanced synaptic plasticity and spatial learning skills by increasing the proteasome activity [62]. SFN attenuated scopolamine-induced deterioration of memory in rats, in association with the hippocampal induction of BDNF and CREB expression and the enhancement of hippocampal synaptic activity [63]. Also, bioactive *R_S_*-GRA-treatment protected against the neuronal functional disruption, restoring dendritic spine count in a Parkinson’s disease model [64]. A study evidenced that Phenethyl ITC can induce cytoskeletal changes [65]. Then, our study confirms the role of ITCs in the modulation of synaptic plasticity and cytoskeleton regulation. Moringin, like other isothiocyanates, is known to activate the TRPA1 ion channel at the concentrations used in this study [66]. *TRPA1* is expressed in motoneurons and oligodendrocytes, and its activation has been linked to neuromuscular symptoms such as cramps and fasciculations, particularly in individuals carrying hyperactive *TRPA1* variants [67,68]. TRPA1 activation, or possibly desensitization, might have contributed to the transcriptional changes we observed, potentially through calcium signaling or oxidative stress pathways, in which it is already known to be involved [69]. Notably, the upregulation of transcription factors such as CREB, which is responsive to calcium and ROS, may point in this direction [70]. Further studies will be needed to explore this potential mechanism. Taken together, these observations suggest that *TRPA1* may represent a relevant modulatory factor in motoneuron physiology. Its responsiveness to electrophilic compounds and its involvement in calcium and oxidative stress signaling indicate that it could play a contributory role in the observed transcriptomic effect, warranting further targeted investigation.

It is important to note that in this preliminary study we used differentiated NSC-34 motor neurons. These cells after differentiation exhibit morphological and physiological characteristics of primary motor neurons, such as long branching processes and neurite outgrowth and the expression of neurofilament proteins. Moreover, differentiated NSC-34 cells also showed the upregulation of AChE and accumulation of synaptophysin in growth cone-like structures [71]. However, these transcriptomic changes should also be verified in primary motor neurons.

## 4. Materials and Methods

### 4.1. Synthesis of the α-CD/MOR Complex

MOR was produced by hydrolyzing Moringa oleifera seeds (cake powder PKM2 supplied by Indena India Pvt. Ltd., Bangalore, India) thanks to the use of myrosinase of GMG. Reverse-phase chromatography was used to purify it. The preparation procedure is detailed in [72,73]. The α-CD/MOR combination has been developed in accordance with [11] and described by Mathiron et al. [24].

### 4.2. NSC-34 Culture, Differentiation, and Treatment

The Cedarlane Corporation, located in Burlington, ON, Canada, provided the NSC-34 cell line. The maintenance medium consisted of DMEM High Glucose enriched with 10% Fetal Bovine Serum (FBS), 1% penicillin/streptomycin, and 1% L-Glutamine (Sigma-Aldrich, Merck KGaA, Darmstadt, Germany). Six-well plates were used to seed NSC-34 cells. At 24 h after seeding, NSC-34 cells were cultured for 5 days using the following medium to induce cell differentiation: 1:1 DMEM/F-12 (Ham), 1% FBS, 1% L-glutamine, 0.5% penicillin/streptomycin, and 1 µM retinoic acid (Sigma-Aldrich, Saint Louis, MO, USA). At the conclusion of the differentiation process, cells were treated with varying concentrations of α-CD/MOR (0.5 µM, 5 µM, and 10 µM) for 96 h. α-CD/MOR was directly dissolved in the medium, and the medium was replaced with fresh medium containing α-CD/MOR every 24 h throughout the duration of the treatment. Based on previous studies, differentiated NSC-34 cells exhibit morphological and physiological characteristics of primary motor neurons (neurite outgrowth and expression of neurofilament proteins, synthesis and storage of Ach) [71].

### 4.3. Library Preparation and Sequencing

After 96 h of treatment with the different concentrations of α-CD/MOR, cells were harvested, and the Maxwell^®^ RSC Simply RNA Cells Kit (Promega, Madison, WI, USA) was used to extract total RNA. Following the protocol, a cDNA library for transcriptome analysis was created using the TruSeq RNA Exome, and sequencing was made using NextSeq 550 instrument (Illumina, San Diego, CA, USA).

### 4.4. Comparative Transcriptomic and In Silico Analysis

The quality score of each base of the runs was checked using The FastQC tool (version 0.11.9, Babraham Institute, Cambridge, UK) was used to analyze the base quality score and Trimmomatic (version 0.40-rc1, Usadel Lab, Aachen, Germany) [74] to cut the adapters from each sequence.

STAR RNA-seq aligner (version 2.7.10a_alpha_220207, New York, NY, USA) [75] was run against the genome of Mus Musculus (version vM28) obtained in GENCODE to map the reads while HTSeq (version 0.13.5) [76] to count the reads. The comparative analysis, performed in R version 4.2.0 (R Core Team) with DESeq2 library version 1.36.0 [77] allowed us to extract genes that were defined as DEGs when the *p*-value corrected using the Benjamini–Hochberg method was lower than 0.05 to remove false-positive genes.

All DEGs were imported in SynGO (30 June 2025). Since SynGO needs human alias, we used the built-in convert tool removing the unmappable genes. Then, the mapped alias DEGs were enriched by SynGO. The overrepresented terms were inspected with AmiGO2 (30 June 2025).

The packages BiomaRt (version 2.58.2), clusterProfiler (version 4.6.2) and org.Mm.eg.db (version 3.16.0) were used to obtain the list of ortholog genes and perform KEGG overrepresentation, and SPIA (version 2.54.0) to show the activation or inhibition of the KEGG pathways. Data manipulation was made using packages dplyr (version 1.1.4) and tidyverse (version 2.0.0). The packages UpSetR (version 1.4.0) and ggplot2 (version 3.5.0) were used to plot data.

All the DEGs from different comparisons were uploaded to STRING databases [78] to inspect the connections across all the proteins and build an interaction network. The networks resulted from STRING were analyzed using Cytoscape v.3.10.3 [79]. We first removed all proteins not involved in the main network, and then we filtered out all the proteins with a betweenness centrality below the 90th percentile.

The Enrichr tool from online web page was used in our experiments to perform the overrepresentation of transcription factors and diseases using the ChEA and DisGeNET database, respectively. The levels of expression of the transcripts in the different brain areas were obtained from the Human Protein Atlas.

### 4.5. Protein Extraction and WTestern Blot

After the 96 h treatments with the different α-CD/MOR concentrations, NSC-34 cells were harvested, and protein extraction was carried out with the NE-PER™ Nuclear and Cytoplasmic Extraction Reagents (Thermo Scientific™, Waltham, MA, USA), according to the manufacturer’s instructions. Bradford assay (Bio-Rad, Hercules, CA, USA) was used to obtain protein concentrations. Thirty micrograms of cytoplasmatic proteins were heated for 5 min at 95 °C and then loaded and resolved using SDS-polyacrylamide gel electrophoresis (SDS-PAGE). Proteins were transferred onto a PVDF membrane (Immobilon-P, Millipore, Burlington, MA, USA). Membranes were blocked with 5% skim milk dissolved in TBS for 1 h at room temperature. Then, membranes were incubated overnight at 4 °C with the following antibodies: synaptophysin (1:10000; Abcam, Cambridge, UK); PSD95 (1:1000; Abcam, Cambridge, UK); βIII tubulin (1:1000; Cell Signaling Technology, Danvers, MA, USA). Membranes were washed with TBS 1×, and incubated with HRP-conjugated anti-rabbit (1:2000; Santa Cruz Biotechnology Inc., Dallas, TX, USA) or anti-mouse antibody (1:2000; ThermoFisher Scientific, Rockford, IL, USA) for 1 h at room temperature. The protein bands were visualized using the Immobilion Forte Western HRP Substrate (Millipore Corporation, Burlington, MA, USA). Protein band acquisition was obtained using the ChemiDoc™ XRS + System (Bio-Rad, Hercules, CA, USA), while protein band quantification was carried out using ImageJ 1.53t software. Restore Western Blot buffer (Thermo Scientific, Meridian, Rockford, IL, USA) was used to strip the membranes, and then they were incubated with GAPDH HRP-conjugated antibody (1:1000; Cell Signaling Technology, Danvers, MA, USA) used as loading controls. The original uncropped blots are available in Figures S5–S7.

### 4.6. Statistical Analysis

Western blot statistical analysis was performed with GraphPad Prism version 10.2.3 software (GraphPad Software, La Jolla, CA, USA). One-way ANOVA test and the Bonferroni post hoc test were used for multiple comparisons among the groups. A *p*-value ≤ 0.05 was considered statistically significant. The results were represented as the mean ± standard deviation (SD).

## 5. Conclusions

In this preliminary study, we found that prolonged treatment (96 h) with α-CD/MOR modulated synaptic genes and pathways in differentiated NSC-34 cells. Moreover, the α-CD/MOR dose of 10 μM is the most effective in promoting the modulation of genes involved in cytoskeleton regulatory pathway, that is associated with synaptic organization and functions. Our study showed that α-CD/MOR also modulated transcription factors associated with synaptic plasticity. Therefore, α-CD/MOR may activate a transcriptional program which modulates actin dynamics and reinforce synaptic plasticity. Interestingly, the genes modulated by α-CD/MOR are involved in various neurological, neurodegenerative, and neuropsychiatric disorders. This result could be of interest because of the potential use of α-CD/MOR as an integrative therapy for improving synaptic plasticity in different synaptopathies associated with cytoskeletal alteration. In the future, these transcriptomic changes should also be verified in primary motor neurons to confirm α-CD/MOR effects.

## Figures and Tables

**Table 1:** Statistically significant gene ontology terms according to SynGO.

GO Term ID	GO Domain	GO Term Name	GSEA Count Background	GSEA Count Input	Comparisons
GO:0045202	CC	synapse	1478	506	CTRL vs. α-CD/MOR_0.5-96_
				551	CTRL vs. α-CD/MOR_5-96_
				639	CTRL vs. α-CD/MOR_10-96_
GO:0098793	CC	presynapse	692	233	CTRL vs. α-CD/MOR_0.5-96_
				258	CTRL vs. α-CD/MOR_5-96_
				282	CTRL vs. α-CD/MOR_10-96_
GO:0098794	CC	postsynapse	911	321	CTRL vs. α-CD/MOR_0.5-96_
				341	CTRL vs. α-CD/MOR_5-96_
				405	CTRL vs. α-CD/MOR_10-96_
GO:0014069	CC	postsynaptic density	348	125	CTRL vs. α-CD/MOR_0.5-96_
				132	CTRL vs. α-CD/MOR_5-96_
				162	CTRL vs. α-CD/MOR_10-96_
GO:0140236	BP	translation at presynapse	51	31	CTRL vs. α-CD/MOR_5-96_
GO:0140242	BP	translation at postsynapse	56	33	CTRL vs. α-CD/MOR_5-96_
GO:0099572	CC	postsynaptic specialization	415	183	CTRL vs. α-CD/MOR_10-96_
GO:0050808	BP	synapse organization	424	195	CTRL vs. α-CD/MOR_10-96_

The list of gene ontology terms that are obtained by gene set enrichment analysis using SynGO. In GO domain column, CC stands for terms associated with cellular component, while BP stands for terms in the biological process category.

## Data Availability

The data supporting the findings of this study are openly available in the NCBI Sequence Read Archive at BioProject, accession number PRJNA1117421.

## Supplementary Materials

The following supporting information can be downloaded at https://www.mdpi.com/article/10.3390/ijms26178220/s1.

## Abbreviations

The following abbreviations are used in this manuscript:

ITCs	Isothiocyanates
GLs	Glucosinolates
GRA	Glucoraphanin
SFN	Sulforaphane
GMG	Glucomoringin
MOR	Moringin
ALS	Amyotrophic lateral sclerosis
AD	Alzheimer’s disease
CD	α-cyclodextrin
CTRL	Control
DEGs	Differentially expressed genes
