# Supplementary figures and images for: α-Cyclodextrin/Moringin Impacts Actin Cytoskeleton Dynamics with Potential Implications for Synaptic Organization: A Preliminary Transcriptomic Study in NSC-34 Motor Neurons
Source: Int J Mol Sci. 2025 Aug 24;26(17):8220. doi: 10.3390/ijms26178220 (PMC12428659; doi:10.3390/ijms26178220)

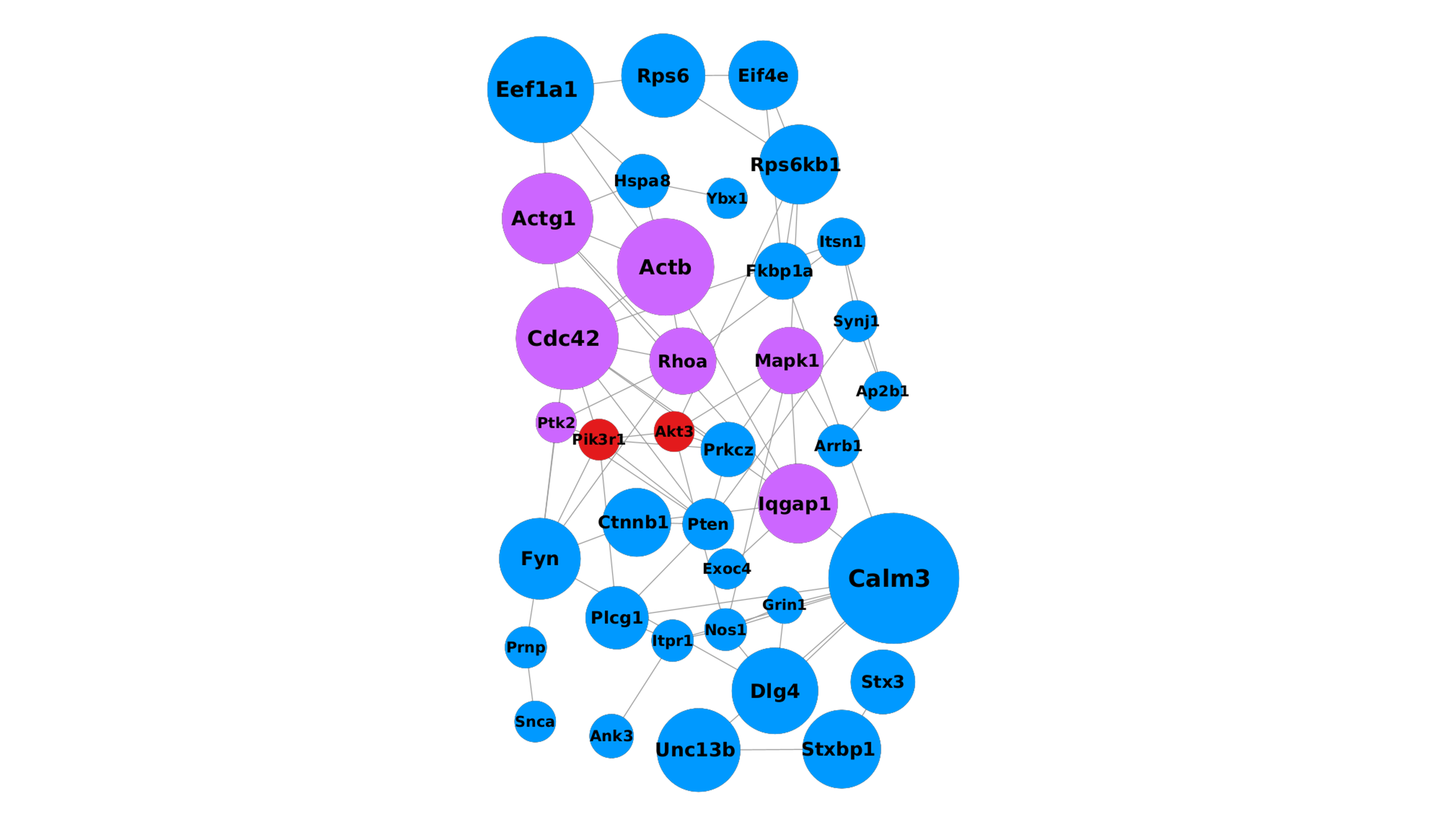

Supplement: Supplementary file 1 [file ijms-26-08220-s001.zip › Figure S1.tif]

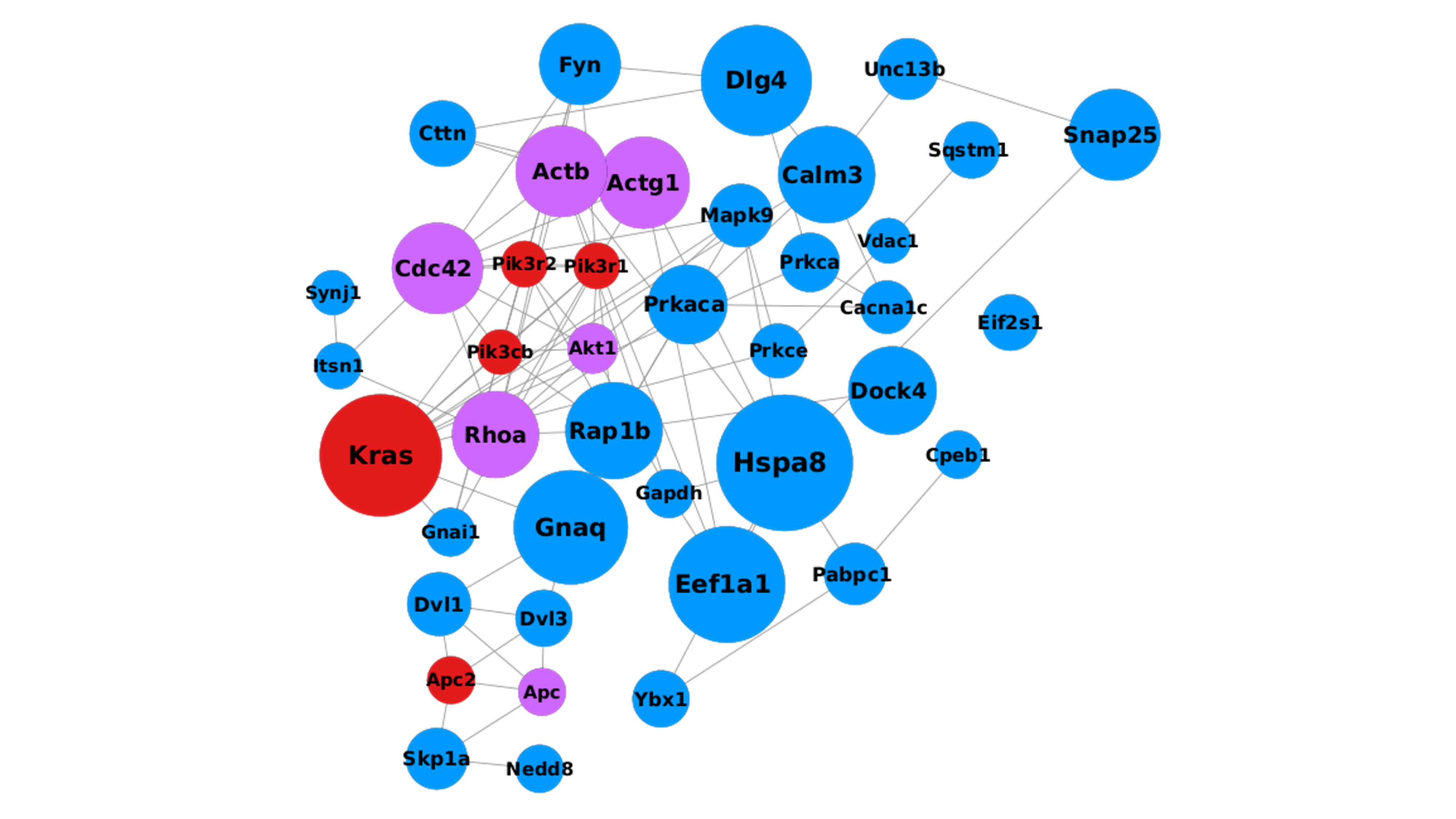

Supplement: Supplementary file 1 [file ijms-26-08220-s001.zip › Figure S2.tif]

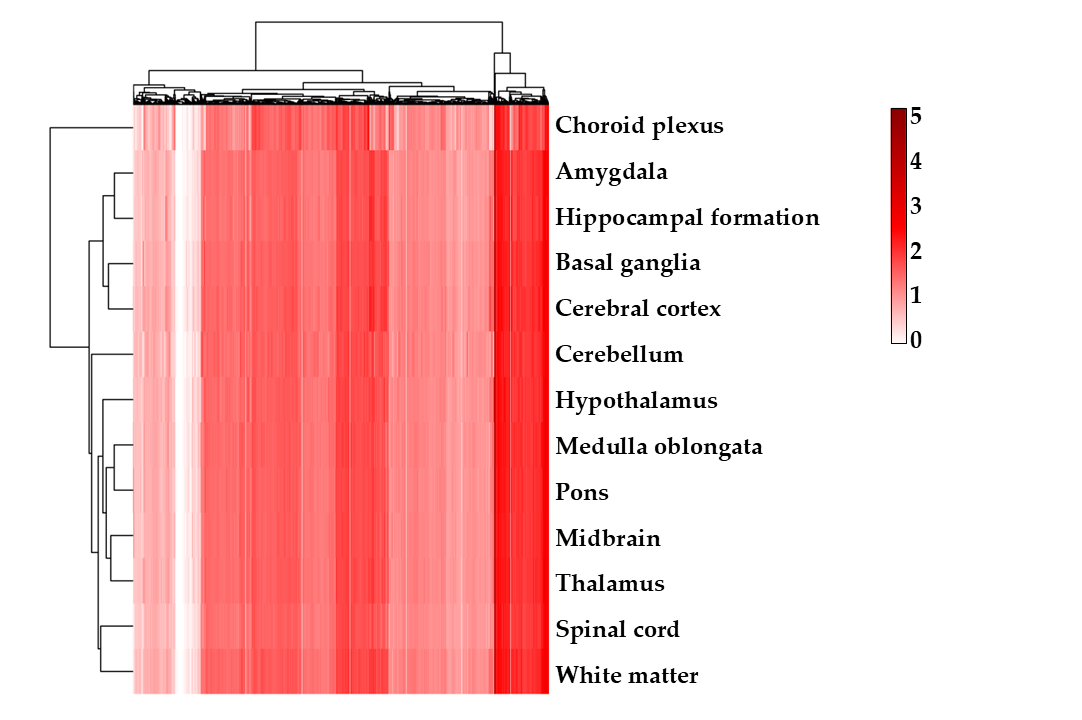

Supplement: Supplementary file 1 [file ijms-26-08220-s001.zip › Figure S3.TIF]

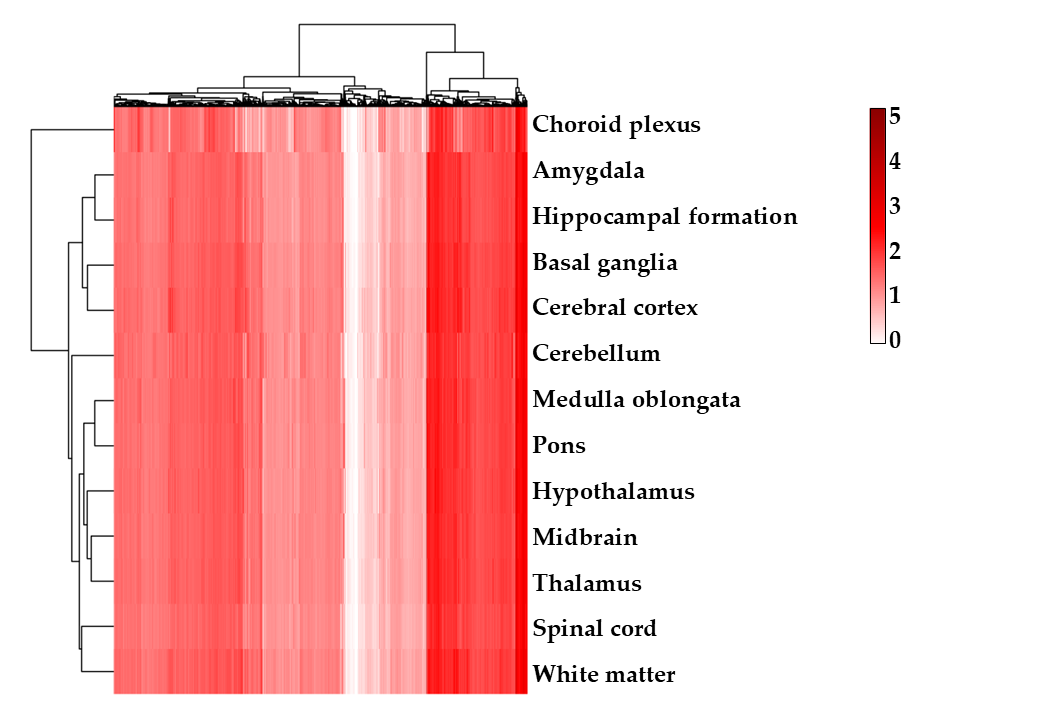

Supplement: Supplementary file 1 [file ijms-26-08220-s001.zip › Figure S4.TIF]

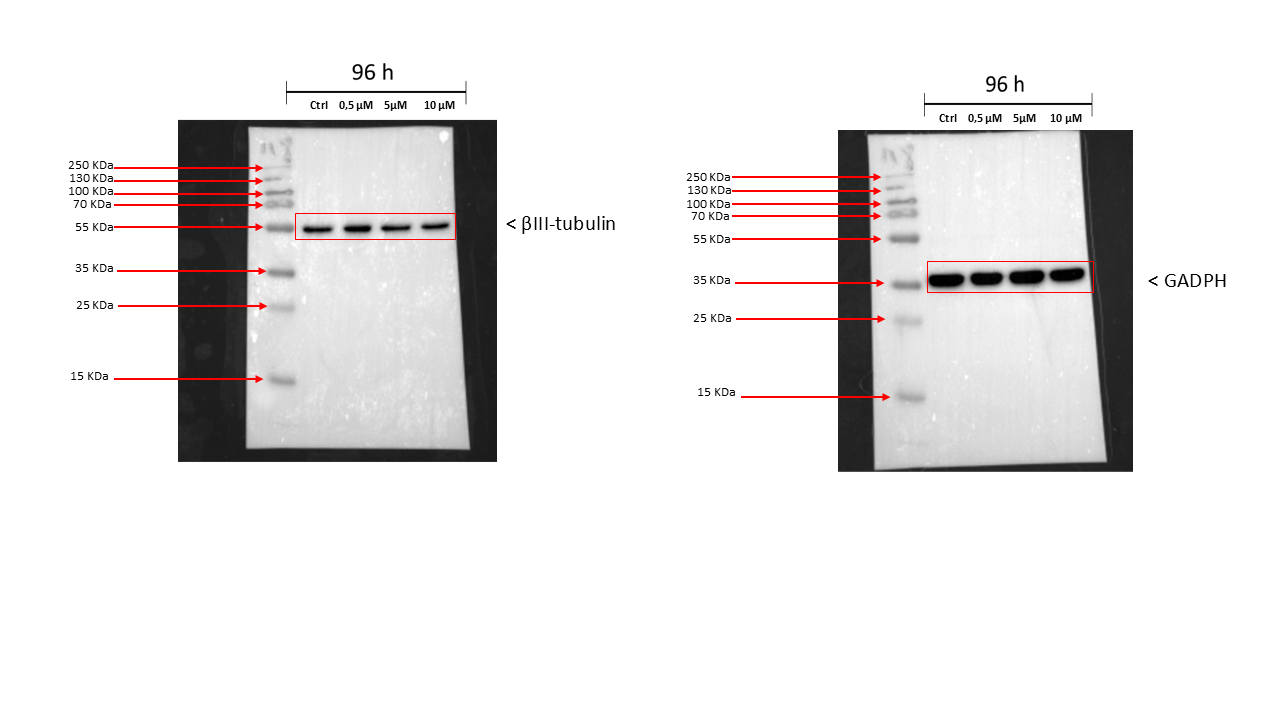

Supplement: Supplementary file 1 [file ijms-26-08220-s001.zip › Figure S5.TIF]

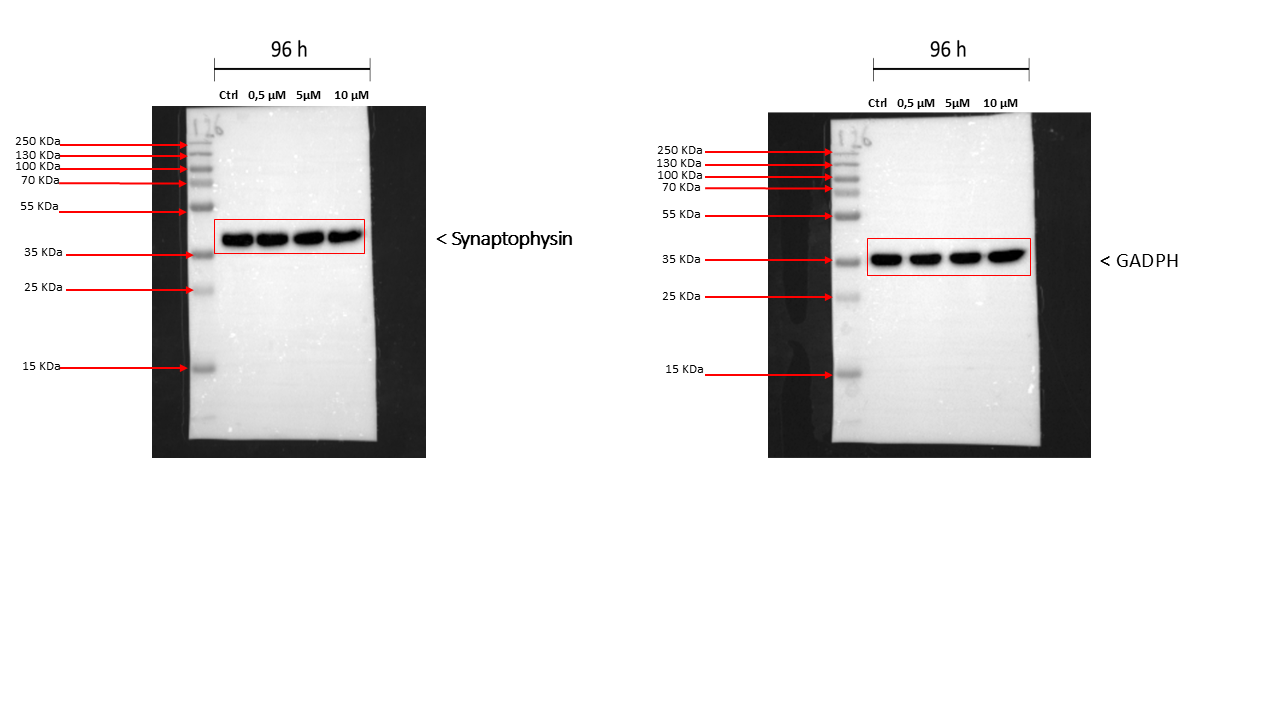

Supplement: Supplementary file 1 [file ijms-26-08220-s001.zip › Figure S6.TIF]

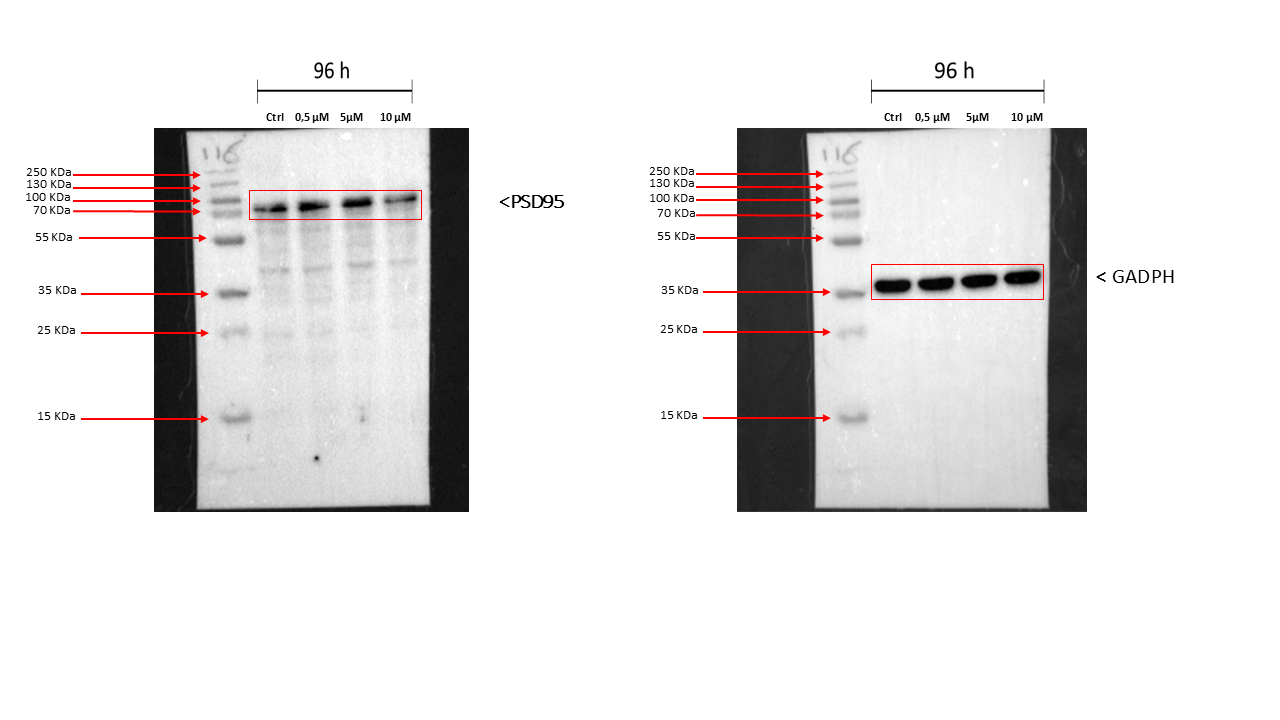

Supplement: Supplementary file 1 [file ijms-26-08220-s001.zip › Figure S7.TIF]
